# Safety of a short-term infusion of fosnetupitant in patients with gastrointestinal and breast cancer: a prospective study

**DOI:** 10.1093/oncolo/oyae223

**Published:** 2024-09-11

**Authors:** Akinobu Nakata, Naoya Hashimoto, Yukiya Narita, Munehiro Wakabayashi, Hiroyuki Kodama, Takatsugu Ogata, Kazunori Honda, Toshiki Masuishi, Hiroya Taniguchi, Shigenori Kadowaki, Masashi Ando, Yuka Endo, Haruru Kotani, Ayumi Kataoka, Masaya Hattori, Akiyo Yoshimura, Masataka Sawaki, Kazuki Nozawa, Isao Oze, Hiroji Iwata, Kei Muro

**Affiliations:** Department of Clinical Oncology, Aichi Cancer Center Hospital, Nagoya, Aichi 484-8681, Japan; Department of Pharmacy, Aichi Cancer Center Hospital, Nagoya, Aichi 484-8681, Japan; Department of Clinical Oncology, Aichi Cancer Center Hospital, Nagoya, Aichi 484-8681, Japan; Department of Clinical Oncology, Aichi Cancer Center Hospital, Nagoya, Aichi 484-8681, Japan; Department of Clinical Oncology, Aichi Cancer Center Hospital, Nagoya, Aichi 484-8681, Japan; Department of Medical Oncology, Osaka International Cancer Institute, Osaka 540-0008, Japan; Department of Clinical Oncology, Aichi Cancer Center Hospital, Nagoya, Aichi 484-8681, Japan; Department of Clinical Oncology, Aichi Cancer Center Hospital, Nagoya, Aichi 484-8681, Japan; Department of Clinical Oncology, Aichi Cancer Center Hospital, Nagoya, Aichi 484-8681, Japan; Department of Clinical Oncology, Aichi Cancer Center Hospital, Nagoya, Aichi 484-8681, Japan; Department of Clinical Oncology, Aichi Cancer Center Hospital, Nagoya, Aichi 484-8681, Japan; Department of Breast Oncology, Aichi Cancer Center Hospital, Nagoya, Aichi 484-8681, Japan; Department of Breast Oncology, Aichi Cancer Center Hospital, Nagoya, Aichi 484-8681, Japan; Department of Breast Oncology, Aichi Cancer Center Hospital, Nagoya, Aichi 484-8681, Japan; Department of Breast Oncology, Aichi Cancer Center Hospital, Nagoya, Aichi 484-8681, Japan; Department of Breast Oncology, Aichi Cancer Center Hospital, Nagoya, Aichi 484-8681, Japan; Department of Breast Oncology, Aichi Cancer Center Hospital, Nagoya, Aichi 484-8681, Japan; Department of Breast Oncology, Aichi Cancer Center Hospital, Nagoya, Aichi 484-8681, Japan; Center for Cancer Genomics and Advanced Therapeutics, Aichi Cancer Center Hospital, Nagoya, Aichi 484-8681, Japan; Division of Cancer Epidemiology and Prevention, Aichi Cancer Center Research Institute, Nagoya, Aichi 484-8681, Japan; Department of Breast Oncology, Aichi Cancer Center Hospital, Nagoya, Aichi 484-8681, Japan; Department of Clinical Oncology, Aichi Cancer Center Hospital, Nagoya, Aichi 484-8681, Japan

**Keywords:** fosnetupitant, CINV, short-term infusion

## Abstract

**Background:**

Fosnetupitant, a neurokinin-1 receptor antagonist, is used to prevent chemotherapy-induced nausea and vomiting (CINV) in patients undergoing highly emetogenic chemotherapy (HEC) or moderately emetogenic chemotherapy (MEC). Previous phase III trials demonstrated the non-inferiority of its 30-minute infusion to fosaprepitant in efficacy and a favorable safety profile.

**Methods:**

This was a single-arm, phase II study to investigate the safety of a 15-minute infusion of fosnetupitant in patients with gastrointestinal and breast cancer. Patients who had received their dose of fosnetupitant in a 30-minute infusion without developing an allergic reaction were eligible and received their next fosnetupitant dose for 15 minutes. The primary endpoint was the incidence of an allergic reaction during the first 15-minutes infusion, and the secondary endpoints were the incidence of injection site reaction (ISR), the incidence of a grade ≥ 3 treatment-related adverse event (TRAE) with fosnetupitant, and complete response (CR) rate.

**Results:**

The study period was from February 17, 2023 to June 20, 2023. In an exploratory analysis, medical records from the end of the study period to December 31, 2023 were retrospectively evaluated to assess the time-saving effect and safety of the short-term infusion of fosnetupitant. Fifty-six patients with gastrointestinal and 14 patients with breast cancer were enrolled, one of whom with breast cancer did not receive study treatment at her own request. No allergic reactions occurred during the 15-minutes infusion. Furthermore, there were no allergic reactions across all 280 short-term injections (Table 1). Additionally, no ISR or grade 3 or higher TRAE were reported. The CR rate was 87.0%.

**Conclusion:**

Short-term infusion of fosnetupitant, administered over 15 minutes, was demonstrated to be safe and effective for patients receiving HEC or MEC (Japan Registry of Clinical Trials Trial ID: jRCT1041220144).

## Teaser

This single-arm, phase II study investigated the safety of a 15-minute infusion of fosnetupitant in patients with gastrointestinal and breast cancer.

**Lessons learned**

- A short-term infusion of fosnetupitant (from 30 to 15 minutes) was safe and feasible without allergic reactions.
- A 15-minute infusion of fosnetupitant is a therapeutic option for patients receiving highly emetogenic chemotherapy or moderately emetogenic chemotherapy ( in clinical practice.

## Discussion

To our knowledge, this is the first prospective study demonstrating the safety of a 15-minute infusion of fosnetupitant. We aimed to evaluate the safety of a short-term infusion of fosnetupitant. Our study met its primary endpoint, with an incidence of allergic reaction of 0% (95% CI, 0%-0.052%) during the first short-term infusion. Furthermore, no allergic reaction was observed during a total of 280 short-term infusions (Table 1). No injection site reaction (ISR) or grade 3 or higher treatment-related adverse event (TRAE) was observed with fosunetupitant administration. Chemotherapy combined with fosnetupitant was highly emetogenic chemotherapy (HEC) vs. moderately emetogenic chemotherapy (MEC) + other in 14 (20.3%) versus 55 (79.7%) patients. The CR rate (no vomiting and no rescue medication use) during the overall phase (0 to 120 hours) rate was 87.0%, similar to the previously reported CR rate. There was no difference in the CR rate for combination chemotherapy regimens (HEC vs MEC + other, 92.9% vs 85.5%, Fisher’s exact *P*-value = .33). These results indicate that short-term infusion of fosnetupitant may be safe and useful. As for the actual time reduction effect, the time reduction per patient in this exploratory study was 60.9 ± 53.6 minutes (mean ± SD). A total of 280 shortened doses resulted in a time reduction of 4200 minutes, suggesting a benefit not only to the individual patient, but also to the medical staff. This allows patients, especially outpatients, to receive chemotherapy with ease and reduces the burden on the medical staff. We believe our results support that a 15-minute infusion of fosnetupitant is a viable treatment option for patients with solid cancers in clinical practice.

**Table 1.** Results of a short-term infusion of fosnetupitant (*n* = 69).

Factors	Results
Average infusion time for first dose of a short-term infusion, mean ± SD, min	15.9 ± 1.4
Incidence of allergic reaction of a short-term infusion, % (95% CI)	0 (0-0.052)
Number of short-term infusions per patient, mean ± SD	4.1 ± 3.6
Total number of short-term infusions	280

## Trial Information

**Table:** 

Disease	Advanced cancer/solid tumor only
Stage of disease/treatment	Metastatic/advanced
Prior therapy	No designated number of regimens
Type of study	Phase II, single arm
Primary endpoint	Safety
Secondary endpoints	Safety and efficacy

## Additional Details of Endpoints or Study Design

Eligibility criteria included the following: 1) Patients with solid tumors who have received fosnetupitant and are scheduled to receive it continuously. 2) The age of the patients on the registration date is 18 years or older. 3) ECOG PS 0, 1, or 2. 4) Patients whose latest test values within 28 days before registration (CTCAE v5.0) meet all of the following. (i) AST(GOT) grade ≤ 1, (ii) ALT (GPT) grade ≤ 1, (iii) total bilirubin grade ≤ 1, 5) patients who are able to comply with the protocol requirements, 6) patients who have voluntarily given written consent after understanding the content of the informed consent document.

Our study aimed for a maximum 13% allergic reaction rate, with α and β errors set at 0.05 and 0.01, and a minimum of 70 patients.

## Drug Information

**Table:** 

Generic/working name	Fosnetupitant
Company name	TAIHO PHAMA
Drug type	Selective NK1 receptor antagonist
Drug class	Gastrointestinal agent
Dose	235 mg per body
Route	IV
Schedule of administration	In combination with palonosetron or granisetron and dexamethasone, 235 mg of fosunetupitant is administered intravenously once on day 1 of anticancer treatment

## Patient Characteristics

**Table:** 

Number of patients, male	24
Number of patients, female	45
Stage	Stage I: 1 (1%), stage II: 6 (9%), stage III: 18 (26%), stage IV: 44 (64%)
Age: median (range)	62.0 (32-85)
Number of prior systemic therapies: median (range)	1st line: 19 (28%), 2nd line: 9 (13%), 3rd line: 6 (9%), 4th line: 1 (1%), ≥ 5th line: 4 (6%), NAC or Adj: 30 (43%)
Performance status: ECOG	0: 59 1: 10 2: 0 3: 0 4: 0
Cancer types or histologic subtypes	Esophageal cancer: 2 Gastric cancer: 14 Small intestine cancer: 1 Colorectal cancer: 39 Breast cancer: 13

## Primary Assessment Method

**Table:** 

Title	Incidence of allergic reaction during a day (0 to 24 hours) when fosnetupitant was administered for the first time for 15 minutes
Number of patients screened	70
Number of patients enrolled	69
Number of patients evaluable for toxicity	69
Number of patients evaluated for efficacy	69
Outcome notes	The incidence of allergic reaction at the first 15-minute infusion was 0% (95% CI, 0-0.052)

## Secondary Assessment Method

**Table:** 

Title	✔Incidence of ISR ✔Incidence of grade ≥ 3 TRAEs ✔Complete response (CR) rate (no vomiting and no rescue medication use) during the overall phase (0-120 hours)
Number of patients screened	70
Number of patients enrolled	69
Number of patients evaluable for toxicity	69
Number of patients evaluated for efficacy	69
Outcome notes	The incidence of ISR was 0% (0/69) No grade ≥ 3 TRAEs were observed The CR rate during the overall phase was 87.0% (60/69)

## Assessment, Analysis, and Discussion

**Table:** 

Completion	Study completed
Investigator’s assessment	Shortened infusion of fosnetupitant is a safe and feasible method

Fosnetupitant, a novel neurokinin-1 receptor antagonist, is used for the prevention of chemotherapy-induced nausea and vomiting in patients who receive highly (HEC) or MEC. In a phase III trial (CONSOLE), fosnetupitant demonstrated non-inferiority to fosaprepitant in overall complete response rate (CR, 75.2%) of emesis and a favorable safety profile.^1^ In phase III trials (CONSOLE and CONSOLE-BC), fosnetupitant was infused over 30 minutes to verify efficacy.^1,2^ Fosnetupitant is administered over 30 minutes in clinical practice. Increased time spent in cancer treatment, “time toxicity,” has been reported to negatively impact a patient’s meaningful survival.^3^ The current practice of administering fosunetupitant over 30 minutes, which is expected to have a low incidence of allergic reactions, may be considered to represent “time toxicity” that could be reduced.

It has been suggested that rapid infusion of drugs may increase allergic reactions.^4^ However, if allergic reactions due to drugs are rare, the risk of allergic reaction due to rapid infusion is considered to be limited.^5^ In clinical trials, fosnetupitant caused allergic reactions in 9 patients (1.8%), one of which was grade 3, while all others were grade 2 or less.^1,2^ Based on these results, the incidence of an allergic reaction to fosnetupitant administration is low at approximately 2%. However, there have been no reports of short-term fosnetupitant administration methods. We hypothesized that short doses administered for 15 minutes should be safe, given the low incidence of allergic reactions.

**Figure 1.**
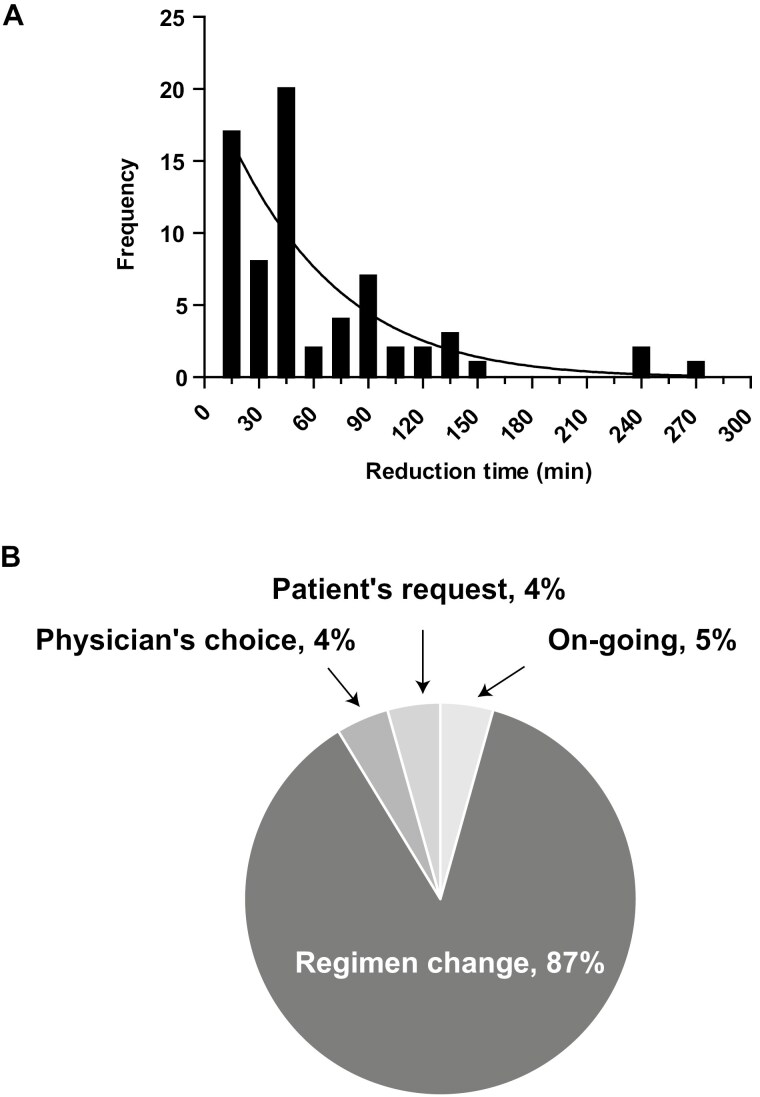
The actual time reduction (*n* = 69). (A) Histogram indicating shortened time per patient. The curve overlapping the histogram shows a normal distribution. (B) Reasons for discontinuation of short-term infusion of fosnetupitant.

The incidence of allergic reactions during the first 15-minute infusion, the primary endpoint, was 0% (95% CI, 0%-0.052%). Furthermore, no allergic reactions were observed after a total of 280 short-term injections. In addition, there were no ISRs or grade 3 or higher adverse events due to short-term infusions. The CR rate was 87.0%, similar to the previously reported CR rate (75.2%). Combination chemotherapy regimens included doxorubicin and cisplatin, cisplatin-based, irinotecan-based, oxaliplatine-based, trastuzumab deruxtecan, 5 fluorouracil and leucovorin, and 14 (20.3%) versus 55 (79.7%) for HEC versus MEC + other. There was also no difference in the CR rates for combination chemotherapy regimens (HEC vs MEC + other, 92.9% vs 85.5%, *P* = .33). Thus, short-term infusion of fosunetupitant was shown to be safe and useful regardless of the combination regimen. A total of 280 short-term infusions, or approximately 4 short-term infusions per patient, were administered. Assuming a time-saving effect of 15 minutes per shortened dose based on the study administration time of 15.9 ± 1.4 minutes (mean ± SD), the overall reducing time was 4200 minutes, or 60.9 ± 53.6 minutes per patient (mean ± SD), leading to reduced time toxicity for the patients (Figure 1A). The majority of discontinuations of the shortened dosing were due to regimen change 60 (87%), with generally good acceptance by both patients and providers (Figure 1B). Fosnetupitant is widely used in combination for HEC and MEC, and short-term infusions reduce the burden on not only the patients with solid tumors, especially outpatients, but also on medical staff.

Our study had several limitations. First, no pharmacokinetic data were available. Second, the inclusion of only patients who had not previously had allergic reactions to fosnetupitant did not allow us to evaluate allergic reactions to the short-term injection in drug-naïve patients. Third, our study included only patients with breast and gastrointestinal cancers. Our results should be validated in a larger sample sufficiently representing other cancer types. However, our study is the first to demonstrate that a 15-minute infusion of fosnetupitant is safe and feasible for patients with solid tumors. This strategy allows patients, especially outpatients, to receive chemotherapy with ease and reduces the burden on the medical staff.

## Data Availability

The data underlying this article are available in Japan Registry of Clinical Trials at https://jrct.niph.go.jp, and can be accessed with jRCT1041220144.
